# Clinical and molecular cytogenetic studies of an unrecognised 22q11.2 deletion in three families

**DOI:** 10.3892/etm.2015.2200

**Published:** 2015-01-21

**Authors:** LINHUAN HUANG, YINGJUN XIE, YI ZHOU, YANMIN LUO, XUAN HUANG, ZHE XU, DANLEI CAI, QUN FANG

**Affiliations:** 1 Fetal Medicine Centre, Department of Obstetrics and Gynaecology, The First Affiliated Hospital of Sun Yat-Sen University, Guangzhou, Guangdong 510080, P.R. China; 2 Division of Cardiac Surgery, The First Affiliated Hospital of Sun Yat-Sen University, Guangzhou, Guangdong 510080, P.R. China; 3 Department of Ultrasonic Medicine, The First Affiliated Hospital of Sun Yat-Sen University, Guangzhou, Guangdong 510080, P.R. China

**Keywords:** 22q11.2 deletion syndrome, phenotypic variability, congenital heart disease, single nucleotide polymorphisms, microarray

## Abstract

The phenotypic variability associated with 22q11.2 deletion syndrome (22q11.2DS) is well known. In the present study, the cases of three unrelated adult patients with chromosome 22q11.2DS and nearly normal features are described, along with their reproductive histories. Chromosomal analysis with fluorescent *in situ* hybridisation and genomic DNA analysis by microarrays were performed, as well as a clinical examination. The three patients were found to possess an identical breakpoint deletion at 22q11.2 by high-density whole-genome single nucleotide polymorphism microarray analysis. The patients had histories of two foetuses/infants with congenital heart defects. The underlying aetiology for the discordance in the phenotype in these patients is discussed. These observations provide additional data useful for patient counselling and guidelines for 22q11.2 clinical screening.

## Introduction

Velocardiofacial syndrome/DiGeorge syndrome (VCFS/DGS) is caused by a 1.5–3-Mb microdeletion of chromosome 22q11.2, and is frequently known as 22q11.2 deletion syndrome (22q11.2DS) [Mendelian Inheritance in Man (MIM) no. 188400/192430]. This syndrome is typically characterised by conotruncal heart defects, a cleft palate, thymic and parathyroid dysplasia with resulting immune defects, hypocalcaemia and learning disabilities (1).

Although 22q11.2DS is the most frequent interstitial deletion in humans, this syndrome presents a wide phenotypic spectrum with >180 clinical manifestations. The estimated prevalence of the syndrome is one in 4,000 live births (2–4); however, the actual occurrence may be higher due to the variation in severity. Individuals present with symptoms on a spectrum from life-threatening to nearly asymptomatic (5,6). The diagnosis can be missed due to subtle dysmorphic facial features. Numerous studies have focused on defining patients eligible for screening (7–9). Agergaard *et al* (7) found that clinical assessment identified fewer than three-quarters of the patients with a 22q11.2 deletion. In excess of one-quarter of patients are likely to remain undiagnosed if genetic tests are not performed on a routine basis. Oh *et al* (8) reported that characteristic facial expressions and a small stature correlated only with 22q11.2 microdeletions. Furthermore, Mikhail *et al* (9) suggested that the recurrent distal 22q11.2 microdeletions do not represent a single clinical entity, and they proposed categorising these deletions into three types according to their genomic position. The 22q11.2 microdeletion types share certain presenting features; however they each have their own unique features and risks. Recently, based on a clinical and dysmorphologic evaluation of 194 individuals and a review of the literature, Monteiro *et al* (10) defined new guidelines for screening the 22q11.2 deletion and divided patients into four groups: Group I, clinical suspicion of 22q11.2DS with palatal anomalies; group II, clinical suspicion without palatal anomalies; group III, cardiac malformations associated with 22q11.2DS; and group IV, juvenile-onset schizophrenia.

In the present study, three previously undiagnosed 22q11.2DS families were described and clinical and molecular cytogenetic studies were performed. The aim of the study was to provide additional data for prenatal counselling and for the clinical diagnosis of 22q11.2DS.

## Subjects and methods

### Patient consent

Ethical approval was obtained for this study from the Ethics Committee of the First Affiliated Hospital of Sun Yat-Sen University (Guangzhou, China). Photographs of the individuals were taken and used for clinical assessments. Samples and photographs from the patients and their families were obtained following informed consent.

### Patient one

A 31-year-old male without underlying disease sought genetic counselling due to adverse reproductive outcomes. The patient and his wife were normal and non-consanguineous, and there was no familial history of congenital malformations. His wife had one foetus with a ventricular septal defect (VSD) and a normal chromosome karyotype analysis; however, the foetus was not tested for the 22q11.2 microdeletion. Two years later, a second foetus exhibited cardiac anomalies (VSD, transposition of conducting arteries and pulmonary artery stenosis), nuchal fold thickening and bilateral renal pelvis separation on an ultrasound scan. Prenatal diagnosis was performed using umbilical cord blood. The results showed that the chromosome karyotype analysis was normal but fluorescent *in situ* hybridisation (FISH) for a 22q11.2 microdeletion (probes for TUPLE1 and N25) indicated that a 22q11.2 microdeletion was present.

**Figure 1:**
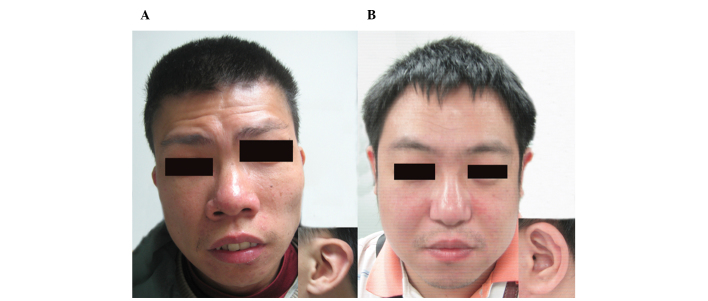
Front and ear views of two patients (A) Patient one: A 31-year-old male with a long face, bulbous nose, broad mouth, thin upper lip and low-set dysplastic ears. (B) Patient three: A 39-year-old male with an bulbous nose and slightly high-set ears with no earlobes.

The father of the patient was 33 years old and his mother was 30 years old at the time of his birth. The patient had a middle school level of education and was frequently ill prior to primary school. His height was 173 cm and his weight was 70 kg (body mass index, 23.4 kg/m^2^). The blood pressure of the patient was 100/60 mmHg and his pulse was 80 bpm. His abdominal examinations were ordinary, and facial features included a long face, pharyngeal abnormalities (two uvulas), bulbous nose, broad mouth, thin upper lip and low-set, dysplastic ears (Fig. 1A).

### Patient two

A 38-year-old male was referred for evaluation as he had a history of two children with congenital heart defects. The patient and his wife were normal and non-consanguineous with no familial history of congenital malformations. The first infant was a term, natural-labour male with a birth weight of 3 kg and without choking-rescue history. When the baby was eight months old, he was diagnosed with tetralogy of Fallot and subsequently passed away. The second child was also a term, natural-labour male. The child had a birth weight of 2.5 kg and no choking-rescue history. A few days after his birth, he was also diagnosed with tetralogy of Fallot. At the age of one year, he succumbed during heart surgery.

The mother of the patient was 39 years old at the time of his birth. The patient has three older brothers and two older sisters, all of whom had a normal pregnancy history. At the age of eight years, the patient caught a high fever (40°C for 24 h) that his parents thought harmed his brain and resulted in a learning disability. No further genetic tests were performed. The height of the patient was 175 cm and his weight was 65 kg (body mass index, 21.2 kg/m^2^). His blood pressure was 110/75 mmHg and his pulse was 85 bpm. The patient had a history of tuberculosis and was often ill before the age of 12. His ears were high set, he had no earlobe and he exhibited auricle reversal (photo not provided).

### Patient three

A 39-year-old male presented with a history of adverse reproductive outcomes. The patient and his wife had two babies with congenital heart defects, were normal and non-consanguineous and had no familial history of congenital malformations. The first child was a term, natural-labour female who was diagnosed with tetralogy of Fallot at birth. The baby succumbed aged nine months. Four years later, a second female was born and diagnosed with pulmonary artery stenosis, atrial septal defect and patent ductus arteriosus at one week old. The baby passed away at 11 months.

The mother of the patient recalled that, during her pregnancy with him, polyhydramnios was found during the second trimester; however no other abnormalities were noted. Until the patient was seven years old, he was susceptible to disease and often caught colds, including tonsillitis. Subsequent to his seventh birthday, the patient’s physique improved. At the time of the study, his height was 162 cm and his weight was 75 kg (body mass index, 28.6 kg/m^2^). The blood pressure of the patient was 150/90 mmHg and his pulse was 80 bpm. Physical features included a bulbous nose and a high-set, small ear with no earlobe (Fig. 1B). With the exception of his hypertension, the health of the patient was unremarkable.

### Molecular studies

#### Conventional cytogenetic analysis and FISH

Peripheral blood samples were collected from the three families, including their spouses and their parents. Foetal blood samples were obtained by cordocentesis. Cytogenetic analysis was performed following the standard harvesting of blood lymphocytes. Metaphase chromosomes were G-banded at 550 bands of resolution.

Metaphase FISH analysis on cultured peripheral blood lymphocytes was performed using a Vysis DiGeorge region probe [LSI TUPLE 1, SpectrumOrange/LSI ARSA SpectrumGreen, fluroescein isothiocyanate (FITC); Abbott Laboratories, Abbott Park, IL, USA] and a Cytocell DiGeorge region probe (TBX1, red spectrum/22qter, green spectrum, FITC; N25, red spectrum/22qter, green spectrum, FITC; Cytocell, Cambridge, UK). A minimum of 20 metaphase cells were assessed under a fluorescence microscope (Leica Microsystems, Wetzlar, Germany).

#### Single nucleotide polymorphism (SNP)-array hybridisation profiling and data analysis

Genomic DNA was isolated from peripheral blood samples using a QIAamp DNA Blood Mini kit (Qiagen, Valencia, CA, USA). DNA concentrations were measured with a NanoDrop spectrophotometer (ND-1000 V.3.1.2; NanoDrop, Thermo Fisher Scientific Inc., Wilmington, DE, USA). The DNA samples (250 ng) were hybridised to CytoScan HD arrays (Affymetrix^®^, Santa Clara, CA, USA) according to the manufacturer’s instructions. The Affymetrix CytoScan HD array includes >2.7 million copy number markers, of which 750,000 are SNPs that can be used for genotyping and 1.9 million are non-polymorphic probes. The Chromosome Analysis Suite software package (Affymetrix) was used for all analyses. Copy number variations (CNVs) were detected by visual inspection of the normalised log2 intensity plots and numerical analysis of the SNP log2 intensity ratios. Copy number changes observed were compared with the CNVs catalogued in the Database of Genomic Variants and the University of Santa Clara in California (UCSC) genome browser. The gene content of the CNVs of interest was determined using the UCSC browser based on the Genome Reference Consortium human genome (GRCH; build 37; http://genome.ucsc.edu/). For putative candidate regions containing at least one gene, each assessment included searches for similar patients in the Database of Chromosomal Imbalance and Phenotype in Humans using Ensembl Resources and PubMed.

## Results

### Conventional cytogenetic analysis

Karyotyping of the cultured lymphocytes from all of the patients indicated a normal karyotype. In addition, the spouses and parents of the patients also had normal karyotypes, based on G-banding techniques with a resolution of 550 bands.

### FISH

The deletion of 22q11.2 was detected by FISH in the three patients. Metaphase FISH analysis of the cultured lymphocytes, using a Vysis DiGeorge region probe and a Cytocell DiGeorge region probe, was used to detect the lack of 22q11.2 signal on chromosome 22, which revealed a deletion at 22q11.2.

### SNP-array analysis

**Figure 2:**
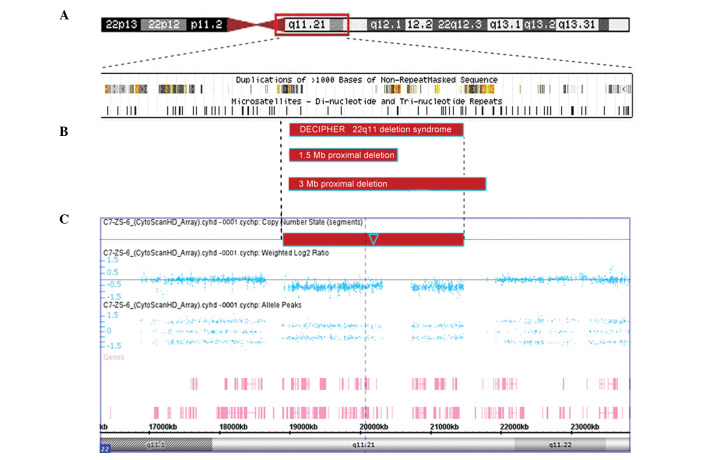
Mapping of a chromosome 22 deletion. (A) Schematic representation of chromosome 22 with dense segmental duplications and microsatellites according to National Centre for Biological Information build 37 (human genome 19). (B) Schematic representation of the 22q11.2 deletion region (LCR22-A to LCR22-H) including the critical region of microdeletion syndrome, as defined in the DECIPHER database (https://decipher.sanger.ac.uk/syndrome/16#genotype/cnv/21/browser) the 3-Mb common typically deleted region or 1.5-Mb DiGeorge critical region found in velocardiofacial syndrome/DiGeorge syndrome. (C) SNP-array genotyping of patients. Whole-genome array-based SNP shows a 2.54-Mb deletion stretching from 18,916,842 to 21,465,659. DECIPHER, Database of Chromosomal Imbalance and Phenotype in Humans using Ensembl Resources; SNP, single nucleotide polymorphism.

The SNP-array narrowed down the deletion size of 22q11.2 for the patients, and revealed that all four patients (three adults and one fetus) shared the same breakpoints (Table I). The molecular details and phenotypic features of the three patients with 22q11.2 deletion are shown in Table I. The deletions were approximately 2.5 Mb, with the identical breakpoint from 22:18,916,842 to 22:21,465,659 (Fig. 2C). This deletion region includes 58 RefSeq and eight Online MIM genes (Fig. 2) encompassing the genes *TBX1, COMT, DGCR2, GP1BB, RTN4R, PRODH, SNAP29* and *SERPIND1*. The results of both the FISH and SNP-array of the parents of the three patients were normal, however the three patients demonstrated a de novo 22q11.2 deletion.

The karyotype of all four patients was therefore 46,XY.ish del(22)(q11.2q11.2)(TUPLE1-,N25-,TBX1-).arr 22q11.2(18,916,842–21,465,659)x1, according to the GRCH 37 (International System for Human Cytogenetics Nomenclature 2009).

## Discussion

The chromosome 22q11.2 region contains eight different chromosome-22-specific low-copy repeats (LCRs) that are known as LCR22s (LCR22-A to LCR22-H). These LCR22s mediate recurrent microdeletions and microduplications by non-allelic homologous recombination (11). Molecular characterisation of the patients in the current study found that the deletion extended from LCR-A to LCR-E, which overlapped with the 3-Mb common typically deleted region or 1.5-Mb DiGeorge critical region (DGCR) observed in VCFS/DGS (Fig. 2).

The 22q11.2 microdeletion was initially detected using FISH analysis for microdeletion/microduplication syndromes during prenatal diagnosis and genetic consultation. This syndrome is usually diagnosed in early childhood in the presence of a typical facial appearance, congenital heart defects, a cleft palate and early-onset hypocalcaemia. By contrast, the patients in the present study were without any cardiac defects and with mild phenotypes (mild developmental delays and dysmorphic features, first diagnosed at the age of >30 years old). Shared traits included two foetuses/infants with a heart defect and decreased childhood immunity. Genomic DNA analysis using an Affymetrix CytoScan HD microarray showed that these patients had identical 22q11.2 deletions of ≥2.5 Mb, including 58 RefSeq and eight Online MIM genes (Fig. 2) encompassing the genes *TBX1*, *COMT*, *DGCR2*, *GP1BB*, *RTN4R*, *PRODH*, *SNAP29* and *SERPIND1*.

Although up to 58 genes are deleted, it is the haplo-insufficiency of the transcription factor *TBX1* that is believed to be the primary contributing factor in this disorder (12–14). T-box transcription factor (Tbx1) belongs to an evolutionarily conserved T-box family of transcription factors, whose expression is precisely regulated during embryogenesis, and appears to regulate the proliferation and differentiation of various progenitor cells during organogenesis that can be clinically affected in this syndrome (15). In addition to other dosage-sensitive genes observed in 22q11.2DS, the incomplete penetration of Tbx1 is the underlying factor for adults without heart defects and with mild dysmorphic features. The low immunity in childhood described by these patients correlated with the defect of cellular immunity in DiGeorge syndrome. Patients with this syndrome present with a broad range of T-cell deficiencies. Foetal thymus transplantation is an effective treatment for reconstituting cellular immunity and normalising the imbalance of regulatory T-cell functions in patients with DiGeorge syndrome (16). Since the patients develop a normal repertoire of mature T-cells, however, the immune defect appears mild in patients surviving the correction of cardiac anomalies (17); therefore, the autoimmune features of 22q11.2DS typically become apparent in early childhood but are rarely diagnosed in adulthood (18,19).

It was found in the present study that the foetus of patient one with a 2.5-Mb deletion presented with severe cardiac defects, whereas his father, with the identical deletion, had no heart defects and only presented with mild developmental delays, bifid uvula and mild dysmorphic features. The other two families did not have the confirmation of genetic detection of their affected foetuses or babies; however, it is suspected that these heart-defect siblings harboured the 22q11.2 deletions transmitted by their fathers.

There are hypotheses regarding the phenotypic variations, such as differences in the size of the deletion, CNVs, epigenetic changes, modifying genetic factors, somatic mosaicism and differences in the intrauterine environment (20); thus, a ‘second hit’ (mutation) in a modifying genetic factor appears more likely for the patients considered in the present study. There are certain mechanisms for the second hit hypothesis, which include replication errors, base changes and additional deletions. According to Stalmans *et al* (21) variations in the gene encoding vascular endothelial growth factor may have the potential to modify the cardiovascular phenotype of hemizygous 22q11.2 deletions. Similarly, a study by Driscoll *et al* (22) described modifiers for palatal phenotypes with this syndrome; as such, the ‘second somatic hit’ is not just restricted to a genetic change at the level of the DNA sequence but may also involve epigenetic changes. It has been demonstrated that the genetic background affects the severity of cardiovascular, thymic and parathyroid anomalies in mice (23,24). The ‘two-hit’ model proposed by Girirajan *et al* (25) stated the requirement for a secondary event during foetal development to cause more severe clinical manifestations. This second hit could be another CNV, a disruptive single-base-pair mutation in a phenotypically relevant gene or an environmental event causing alterations to the phenotype or deletion size, as was observed in the patients from the present study. The two-hit model additionally helps to explain the underlying phenotypic variability that has been reported for several recurrent microdeletions. The majority of second hits are likely to be undetectable, even using high-resolution arrays. Re-sequencing the whole genome, however, may reveal a number of additional contributing loci (20,26).

High-density, whole-genome SNP arrays have an important advantage over conventional karyotyping in microdeletion/microduplication detection, microarray-based copy number analysis and genotype analysis. The resolution is affected by the genomic distance between the probes and their size. Molecular karyotyping provides information directly associated with the physical and genetic map of the human genome, and microarrays enable the identification of small CNVs with a high accuracy. Commercially available arrays are an invaluable tool for the diagnosis of patients presenting with intellectual disabilities and/or multiple congenital abnormalities (27). Array-comparative genomic hybridization or SNP arrays have been demonstrated to represent a cost-effective alternative to multiple FISH testing for the identification of genomic imbalances (28).

In conclusion, three adult cases of 22q11.2DS with mild dysmorphic features have been reported in the present study. Although a range of autoimmune features associated with 22q11.2DS have been described, the condition typically becomes apparent in early childhood and is rarely diagnosed in adulthood. It is worth clinicians considering the diagnosis of 22q11.2DS in an adult patient with a past medical history of compromised immunity, and it is necessary to carry out DNA microarray analysis on couples who have had recurrent abnormal pregnancies to exclude the microdeletion/microduplication syndrome in patients without severe abnormalities or with normal phenotypic manifestations.

## Figures and Tables

**Table I:** Molecular details and phenotypic features of individuals with a 22q11.2 deletion.

Patient	Deleted band	Start site; stop site (bp)^b^	Size (Mb)	Origin	Age (years)	DD	SD	HD	Velopharyngeal abnormalities	Facial dysmorphology	Others
1	22q11.21	18,916,842; 21,465,659	2.549	Dn	31	Mild	+	-	Two uvulas	Long face, bulbous nose, broad mouth, thin upper lip, low-set dysplastic ears	-
1^a^	22q11.21	18,916,842; 21,465,659	2.549	Pat	Fetus	Unk	Unk	VSD TCA, PAS	-	Unk	Unk
2	22q11.21	18,916,842; 21,465,659	2.549	Dn	38	Mild	+	-	-	High-set ears with no lobes, auricle reversal	Two fetuses with congenital heart defect delivered
3	22q11.21	18,916,842; 21,465,659	2.549	Dn	39	No	+	-	-	Bulbous nose, high-set ears with no lobes	Hypertension

Patient 1^a^ was the second child of patient 1. ^b^National Centre for Biotechnology Information 37/human genome 19. Dn, de novo; Pat, paternally inherited; Unk, unknown; DD, developmental delay; SD, speech delay; HD, heart defect; VSD, ventricular septal defect; TCA, transposition of conducting arteries; PAS, pulmonary artery stenosis.

## Abbreviations

22q11.2DS: 22q11.2 deletion syndrome
SNP: single nucleotide polymorphism
FISH: fluorescent *in situ* hybridisation
VCFS: velocardiofacial syndrome
DGS: DiGeorge syndrome
VSD: ventricular septal defect
CNV: copy number variation
LCR: low-copy repeat
DGCR: DiGeorge critical region
